# The Quiet Eye Period and its Relationship With Task Complexity: Is the Ceiling Effect an Indicator of Expertise?

**DOI:** 10.1177/00315125251388328

**Published:** 2025-10-15

**Authors:** Harrison K. Leivers, Kjell N. van Paridon, Peter M. Allen, Oliver R. Runswick, Matthew A. Timmis

**Affiliations:** 1 Cambridge Centre for Sport and Exercise Sciences (CCSES), 2369 Anglia Ruskin University, Cambridge, UK; 2 Vision and Hearing Sciences Research Centre, 2369 Anglia Ruskin University, Cambridge, UK; 3 Department of Psychology, Institute of Psychiatry, Psychology and Neuroscience, King’s College, London

**Keywords:** quiet eye, task demands, expertise, golf, putting

## Abstract

**Background:** The quiet eye (QE) period is defined as the final fixation prior to movement initiation for a minimum period of 100 ms. In golf the QE period continues beyond the ball being struck and typically lasts approximately 2-3s. Longer total-QE, pre-QE (pre-ball contact), and post-QE (post-ball contact) are associated with more complex tasks and higher-skilled golfers. However, excessively long QE durations are most likely counterproductive. **Purpose:** We aimed to use an in-situ design to investigate how task complexity affects QE duration in thirty golfers (10 Sub-elites, 10 Intermediates and 10 Novices). **Data Collection:** Participants performed four shots from increasing distances (greater task complexity). Total-QE, QE-pre, and QE-post were measured. **Results:** Novice total-QE indicated a significant moderate linear relationship with complexity, whereas no relationship was observed in intermediate and sub-elites. Novice QE increased by 53% between the simplest and the most complex condition. **Conclusions:** A ceiling effect may be a discriminating factor between skilled and less-skilled golfers, which could suggest that the mechanisms underpinning the QE in higher-skilled golfers are independent of task demands. The consistent QE durations observed in sub-elite golfers may imply that this period is used to parameterise the optimal movement variant or that this group did not find the most complex task more effortful. Novice golfers were more sensitive to alterations in tasks demands, which may indicate that the QE period reflects a higher cognitive load, explaining a linear increase in QE duration. A ceiling effect may indicate a difference in the purpose of QE between skilled and less skilled golfers.

## Introduction

The quiet eye (QE) period is considered a key visuomotor measure for successful performance within self-paced aiming tasks (Wilson & Pearcy, 2009). QE is defined as the final fixation prior to movement initiation that is located on a specific location or object for a minimum of 100 ms (Vickers, 1996). In golf putting the final fixation typically occurs on the ball prior to task execution and continues beyond the ball being struck, approximately lasting 2000-3000 ms (Carey et al., 2024). In closed-skill aiming tasks the QE period is sensitive to contextual demands (Runswick et al., 2021), complexity (Causer et al., 2017; Walters-Symons et al., 2017), anxiety (Giancamilli et al., 2022; He et al., 2024) and expertise (Klostermann et al., 2014; Williams et al., 2002). The mechanisms underpinning QE are highly debated (see Gonzalez et al., 2017) and typically fall into two categories, termed outward or inward functions (Harris et al., 2023). Outward functions are those that imply the QE period reflects the processing of visual cues which informs action planning and execution, whereas inward functions suggest that QE is reflective of the allocation of cognitive resources and processes (focussing attention/motor planning) (Gallicchio & Ring, 2020).

The QE phenomenon is considered the link between the visual and attentional networks. QE involves a period of ocular stillness that directs attention to a task. During the QE period, key regions of the brain involved in visual attention and motor planning are activated, including the occipital lobe, frontal eye fields, dorsolateral cortex and posterior parietal cortex (Gonzalez et al., 2017). Additionally, the hippocampus and amygdala are engaged, which indicates the role of memory and emotional regulation (Vickers, 2016). Through suppression of the oculomotor system, the QE period allows attention to be directed to relevant task information, minimising distraction by avoiding peripheral (task-irrelevant) locations (Gonzalez et al., 2017). Once visual information has registered, the dorsal attention network (DAN) and the ventral attention network (VAN) are activated (Vickers, 2016). The primary focus of the DAN is to focus attention on specific locations in space alongside sustaining intentions generated internally; this system is also responsible for blocking anxiety-provoking thoughts that may intrude from the VAN system (Vickers, 2016). Alternatively, the primary purpose of the VAN is to direct attention to unexpected stimuli, within this system, the hippocampus distributes memories and activates the amygdala to aid emotional regulation (Lebeau et al., 2016). By activating the DAN at the expense of the VAN, the QE period increases the focus of attention and protects against irrelevant thoughts and emotions, consequently allowing QE to act as a mental buffer and to allow mental readiness to be attained (Lebeau et al., 2016). Researchers propose various functional interpretations that suggest that visual processing or allocation of cognitive resources (focusing attention/motor planning) occur during the QE period (Gonzalez et al., 2017; Lebeau et al., 2016; Vickers, 2016).

The visual hypotheses (Gallicchio & Ring, 2020) suggests that experts collate greater quantities of visual information and processing this information is reflected in an extended QE period (Williams et al., 2002); this hypothesis suggests that the QE period is directly reflective of the complexities of one’s surroundings and that wherein more visually complex environments the QE is likely to extend, as the demands and processing time are directly related. Alternative hypotheses suggest that QE periods represent the allocation of cognitive (attention) resources to motor planning to ensure optimal execution of motor skills. These hypotheses assume that visual information is collected and processed during the preparatory phase (prior to QE) (Gallicchio & Ring, 2020), as skilled golfers verbalise more planning and information-gathering statements than their less skilled counterparts (Carey et al., 2017; Whitehead et al., 2016). Such hypotheses are the inhibition hypothesis (Klostermann et al., 2014), sensorimotor programming (Causer et al., 2017), and the postural-kinematic hypothesis (Gallicchio & Ring, 2020). The inhibition hypothesis suggests QE primarily contributes to superior performance through attention allocated to task-relevant cues and is associated with inhibition of sub-optimal movement variants during preparation and execution (Gonzalez et al., 2017; Klostermann et al., 2014). When considering specific phases of QE, Causer et al. (2017) proposed that QE pre-execution (movement initiation to ball being struck) represents response selection and online control whereas QE post-execution (from the ball being struck to the end of the fixation) involves the use of visual and/or proprioceptive feedback to adjust movements after initiation. This theory proposes that in actions where individuals know they will receive visual feedback, vision is predominantly utilised during the movement rather than the planning phase (pre-programming), to ensure optimal movement execution (Hansen et al., 2006). Similarly, the postural-kinematic hypothesis suggests that longer QE contributes to better performance through improvements in technical kinematics, such as improved postural stability that results in stillness of the trunk, limbs, head and eyes (Gallicchio & Ring, 2020). Alternatively, it has been suggested that the postural-kinematic and visual processing hypothesis are linked, with this period imperative for stabilising eyes and posture alongside a period of exploration of visual information previously acquired through holding a visual image which supports the implementation and development of an action during the QE period (Jacobson et al., 2021).

All hypotheses have a supporting rationale that could apply to athletes at different phases of the motor learning process; therefore, one hypothesis is unlikely to apply to all and the purpose of QE is likely to be task dependent (Harris et al., 2023). These conflicting suggestions have resulted in studies concurrently analysing neural activity during the QE period (Carey et al., 2024; Gallicchio & Ring, 2020; Mann et al., 2011). This approach measures neural activity within the regions of the brain that control eye movements (Gonzalez et al., 2017), therefore offering insights that typical eye-tracking approaches are unable to explore. For instance, QE and cortical activity during the preparatory phase indicated that higher-skilled golfers allocated more resources to visual-spatial processing of the task over conscious movement-related processing (Mann et al., 2011), potentially due to training adaptations. Conflicting findings suggest that cortical activity provides stronger support for the postural-kinematic hypothesis as participants performed less visual processing moments before, during and after putting the ball (Gallicchio & Ring, 2020). Moreover multi-measure study designs have also supported that the QE represents movement planning through a greater suppression of beta (thought to indicate motor-related processing) and theta (measure of attention) activity (Carey et al., 2024; Fang et al., 2022), which may corroborate the inhibition hypothesis and imply QE represents response selection rather than processing visual information or impacting postural stability. However, this hypothesis can only be valid for skilled golfers, as less-skilled golfers are unlikely to have the experience or ability to select from multiple movement variants. These studies primarily focussed on identifying the underlying mechanisms of QE, they did not assess the impact of tasks demands and how this may alter across expertise.

Regardless of the mechanisms at play, the importance of QE for performance has been evidenced through longer QE durations being related to successful outcomes (Causer et al., 2017; Williams et al., 2002). Therefore, interventions elongating the QE period have been designed to accelerate motor learning and skill acquisition (He et al., 2024; Vickers, 2016; Vine et al., 2011). Golfers who underwent QE training self-reported a two-stroke competitive putting improvement post-intervention (Vine et al., 2011). QE training has also been shown to improve putting performance when under pressure, golfers demonstrated a 5% increase in successful putts compared to a 1% increase in those who received technical interventions (He et al., 2024). However, in both studies, QE was not measured within a golfer’s typical environment, nor were task complexity manipulated across expertise, both of which would increase task demands. Interventions aiming to extend the QE period have displayed positive influences, however, there is limited evidence of what QE duration is most beneficial to performance. Although it has been suggested that there is likely an optimal or maximal QE duration that positively impacts performance (Janelle et al., 2000), this period remains unknown. In a throwing task, Klostermann et al. (2018) highlighted that longer QE periods do not necessarily correspond to performance improvements, and highlighted 3000 ms as the optimal QE duration and extending beyond this period does not benefit performance.

Extended QE periods have been associated with more complex aiming tasks as these scenarios require a longer time to set and attune the parameters of the movement (Lier, 2011; Walters-Symons et al., 2017). As the cognitive demands of a golfer's natural environment are heightened compared to an in-laboratory task (Shaw et al., 2021), it is possible that reported QE durations (0.86-2.2s (Carey et al., 2024; Causer et al., 2017; He et al., 2024; Klostermann et al., 2014; Walters-Symons et al., 2017)) underrepresent QE within a naturalistic environment. QE is also sensitive to contextual demands (putting for a tie, win or practice conditions) (Runswick et al., 2021), and it is likely that environmental factors such as wind, imperfect putting surfaces and other environmental distractions would impact a golfer's QE. However, in golf, research has manipulated target size, putter size, shot length (Causer et al., 2017; Walters-Symons et al., 2017) and slope (Klostermann et al., 2014; Lier et al., 2010; Wilson & Pearcy, 2009), despite all conditions reducing performance, longer QE periods only occurred when shot length increased, highlighting that complexity and QE's relationship may not be linear. Should the underlying mechanisms of QE be related to visual information processing, then QE would likely extend with task demands. Alternatively, if movement-related processing underlies the period, QE may be independent of task demands, particularly if skilled athletes are more efficient. Importantly, the aforementioned research (Klostermann et al., 2014; Lier et al., 2010; Walters-Symons et al., 2017) recruited skilled golf players (of note, Wilson & Pearcy, 2009 did not provide an indication of their participants’ skill level). Causer et al. (2017), however, recruited novice golfers. As golfers are yet to be assessed in a real-world environment, it cannot be assumed that the laboratory-based findings transfer to a real-world environment (Yeoman et al., 2020), and observation within a naturalistic environment could provide nuanced differences between experienced and novice golfers. Therefore, QE must be assessed in a representative environment to provide ecologically valid and robust findings (Kihlstrom, 2021).

The attentional and postural fatigue which may result from prolonged fixation prior to executing the shot/putt in golf and its subsequent impact on performance has led to the suggestion that an optimal window for gaze control may exist (Klostermann et al., 2018; Vickers, 2007). The notion that QE may have a linear relationship has been questioned (Janelle et al., 2000), indeed, a threshold value likely exists whereby further increases in QE length will not be beneficial and possibly contribute to performance deterioration (Klostermann et al., 2018). The relationship between QE and athletic performance may resemble an inverted U, with an optimal duration of 3000 ms (Klostermann et al., 2018). Further, since experts have developed an efficient gaze control strategy (Vickers, 1992), expert preparation periods are more effective and efficient, and they likely avoid excessively long QE periods, even in challenging tasks. In contrast, novices may display a linear increase in QE duration, which is observed as a function of task complexity, leading to excessive QE periods. This, however, is yet to be directly established in the literature. An excessive QE period would lead to motor control processes being harmed and cause the interruption of the shielded pursuit of an action, at which point the performance benefits associated with QE would cease to exist, leading to performance being negatively impacted (Klostermann et al., 2018). What remains unclear is whether the previously observed differences in QE length between novice and sub-elite performers will only be apparent at less complex tasks, and when task complexity increases, if these differences will cease to exist.

The inhibition hypothesis has proposed that the underlying mechanisms of the QE period are movement-related and independent of the visual information collated. Therefore, if this hypothesis is applicable, the QE duration would be independent of task complexity, leading golfers to implement a consistent QE period irrespective of complexity. It is also plausible that if a different trend is observed in less skilled golfers, this may indicate that the purpose of the QE period is a function of expertise and that the underlying mechanisms are different at contrasting stages of the development process. Moreover, an optimal period of 3000 ms has been proposed (Klostermann et al., 2018), which is longer than QE periods reported from the in-laboratory studies assessing golf putting (Carey et al., 2024; Causer et al., 2017; He et al., 2024; Klostermann et al., 2014; Walters-Symons et al., 2017); therefore, it is pivotal to identify whether laboratory study designs have underrepresented the duration of the QE period. Through recruiting novice, intermediate and sub-elite golfers, the current study aimed to examine the impact of expertise and task complexity on the QE period on the golf course rather than in a laboratory. As it has been proposed that skilled golfers likely process visual information during the green reading phase (Carey et al., 2024; Gallicchio & Ring, 2020), for our primary hypothesis we anticipated a linear relationship between QE and complexity in novice and intermediate but not sub-elite golfers. This would result in total-QE, pre-QE, and post-QE durations increasing with task complexity in novice and intermediate golfers but not sub-elite golfers. This would support an optimal QE duration (Klostermann et al., 2018) and the inhibition hypothesis (Klostermann et al., 2014). We also hypothesised that sub-elite and intermediate golfers would perform better than novices and that the sub-elite group would perform better than intermediate golfers.

## Methods

### Participants

The sample size was determined through the use of *a priori* power analysis, sample size power calculation (G*Power 3.1, Dusseldorf (Faul et al., 2007)) to determine a medium effect (f = 0.25) for a within-between interaction between skill level and shot complexity. Using 0.05 alpha (α); power (1-β) of 0.80 across four repeated measures (conditions) with a moderate correlation (0.50) between measures required a minimum sample size requirement of 30. A meta-analysis revealed a large expertise effect on QE, however, within studies that compared absolute QE (rather than relative durations), and within-individual comparisons, reported moderate effects (Lebeau et al., 2016). As we planned to assess absolute durations through between-within interaction through recruitment of sub-elite golfers (rather than elite. e.g., professional athletes) we sought to detect a medium effect. Following institutional ethical approval, 30 male participants (age, 30.93 ± 15.39yrs; mean ± SD) volunteered to participate and were categorised into three groups based on golfing ability. Novices (n = 10, aged 23.50 ± 6.23yrs) had minimal golf experience. Intermediate golfers (n = 10, aged 34.00 ± 4.51yrs) had a handicap of 15.60 ± 4.51 (range 13-20) with 13.70 ± 15.92yrs of experience and typically played 1.90 ± 0.83 rounds per week. Through the use of an expertise calculation the final group were defined as sub-elite (Swann et al., 2015). The sub-elite golfers (n = 10; <5 handicap, aged 35.30 ± 3.10yrs) had a handicap of 3.10 ± 1.04 with 20.00 ± 17.81yrs of experience and typically played 3.2 ± 1.4 rounds per week.

### Protocol

**Figure 1.**
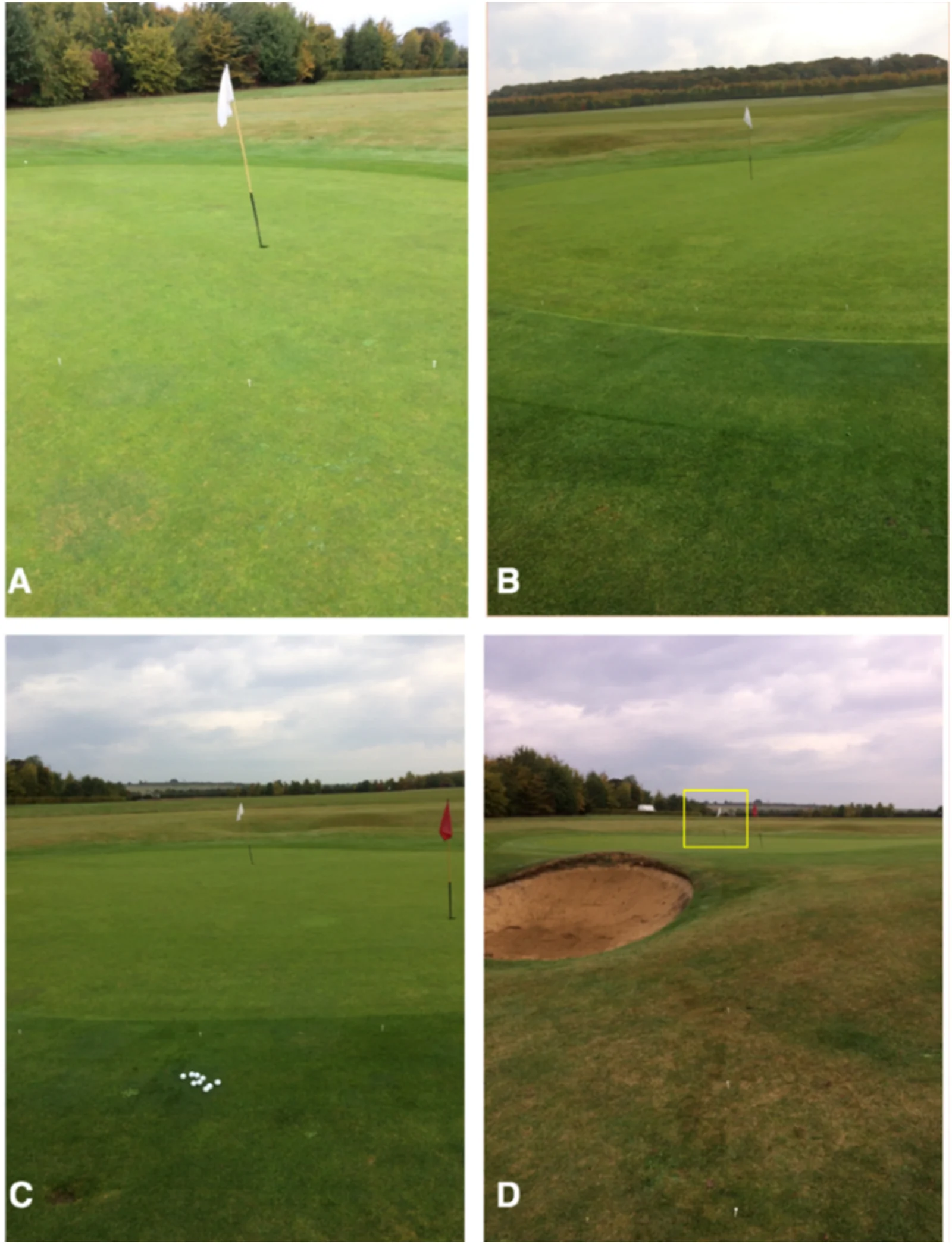
Participants Were Aiming at the White Flag in all Tasks. (A) The 3 m Putt. (B) The 10 m Putt. (C) The 12 m Chip. (D) The 28 m Chip (Yellow Box Outlining). The Flag was Removed when Participants Performed Shots A, B and C

On the same green and under experimental conditions (wearing a SMI iViewETG head-mounted mobile eye tracker), participants warmed up by hitting 16 shots to alternative targets (not holes) on the green, ensuring no prior knowledge of the data collection tasks. The number of warm-up shots was similar to those used in laboratory settings (Carey et al., 2024), in comparison, participants were offered additional warm-up shots as this allowed them to familiarise themselves with the in-situ environment. This allowed participants to familiarise themselves with the conditions (e.g. green speed) and become accustomed to wearing the mobile eye-tracker (additional detail of eye tracker below). In a block randomised study design, participants performed two putts (3m Wilson & Pearcy, 2009) and 10m), and two chip shots (12m (‘bump and run’ shot) and a 28m ‘lobbed shot’). Three shot locations were indicated by tee markers at each distance (see Figure 1). This ensured that the same shot was not performed successively and required participants to complete their preparatory phase each time. The purpose of multiple shot locations at each distance was to avoid over-familiarity, which could lead to a learning effect. Following a shot, participants were instructed to move to a different tee marker and restart their routine. Three shots were performed at each tee marker, with nine shots at each distance (totalling thirty-six trials), QE studies in golfers has ranged from 18-140 shots (Carey et al., 2024; Gallicchio & Ring, 2020; Mann et al., 2011; Runswick et al., 2021). However, we assessed thirty-six shots as this a more accurate representation of the number of putt and chip shots played in a competitive environment whilst providing multiple trials at each distance, which increased the reliability and validity of our findings whilst avoiding fatigue that may impact performance and gaze behaviours. Participants were asked to treat each shot as if participating in a competition. As per (Pelz & Frank, 1999), the researchers explained and demonstrated a ‘bump and run’ shot and a ‘lobbed shot’ to ensure that novice golfers could perform the shots being investigated.

### Apparatus

The study was conducted on outdoor practice facilities at a golf club. The golf hole was regulation size (108 mm diameter). Novices were provided with golf clubs relevant to the shot required; a pitching wedge (Z745, Srixon Golf, Japan) was used for the 28m Chip, 8 Iron (Z745, Srixon Golf, Japan) for the 12m Chip and a Putter (Golo 5 Scotty Cameron putter, Titleist, USA) for putts from 3m and 10m. To our knowledge, no study has directly examined shot selection’s impact on gaze behaviours, although it may impact visuomotor control. Despite this, club design does not impact the implementation of QE, as a result, we did not anticipate this to impact the QE duration of participants (Allen et al., 2024). Therefore, intermediates and sub-elites used their own golf clubs but were instructed to select the same clubs as the novices used (i.e., putter, 8 iron, and wedge). This standardisation ensured comparable shot types across groups and distances, minimising the possibility that any differences observed could be attributed to shot variation rather than gaze behaviours. Pro V1x, Titleist, USA golf balls were used for all shots.

A SMI iViewETG head-mounted mobile eye tracker (SensoMotoric Instruments Inc, Warthestr; Germany, Ver 1.0) recorded eye movements, sampling at 30 Hz. The visual scene was recorded through one high-definition camera (24 Hz), and two infrared cameras enabled eye movements to be recorded. A mini laptop (Lenovo X220, ThinkPad, USA) running iView ETG (Ver. 2.0) software recorded the visual data; this was positioned in a backpack worn by the participant. A three-point calibration was completed, verifying the point of gaze before testing, and every ninth trial was checked. The system’s spatial resolution was 0.1°, with gaze position accuracy of ±0.5° (Timmis et al., 2014).

### Data Analysis

Point of gaze data from the eye tracker was analysed offline using SMI BeGaze software (version 3.4; SensoMotoric Instruments) and was analysed frame-by-frame. Tracking ratio (percentage of time eye movements were measured) of <80% were excluded (Vansteenkiste et al., 2014). The following dependent variables were analysed.

#### Trail Duration

Trial duration was defined as the elapsed time between trial initiation and the end of the trial. Trail initiation occurred when participants adopted a stationary position and grounded the club head behind the ball. The end of the trial was deemed to be when the gaze deviated by 1° of visual angle for more than 100 ms from a participant’s final fixation (the ball). We did not assess visual search behaviours within the preparatory phase, consistent with recent QE investigations (Carey et al., 2024; Gallicchio & Ring, 2020; Walters-Symons et al., 2017). Due to the repeated measures study design, we felt that this might impact the application of the preparatory phase, as previous studies have typically assessed golfers within protocols where they were exposed to the next shot for the first time before cognitively processing information and actioning a motor response (Shaw et al., 2021; Whitehead et al., 2016). Additionally, due to the differences observed in pre-shot routines across participants, we could not reliably standardise the preparatory phase to ensure fair comparison. Previous research has highlighted that higher-skilled golfers extract additional information, which has been indicated through more verbalisations (Carey et al., 2017; Whitehead et al., 2016) and visual search strategies that include fewer fixations of longer durations (Mann et al., 2007, 2016); therefore we did not feel this would provide any further contribution to the literature.

#### Performance

*Shot success:* A successful putt was recorded when participants putted or chipped the ball in the hole. A radial error (RE) was recorded when putts were unsuccessful. RE is defined as the euclidean distance the ball finished from the hole in metres (m) (Klostermann et al., 2014).

##### Quiet Eye

*Pre-QE:* This was the elapsed period between the start of the final fixation (QE-onset) and when the ball was struck. Pre-QE commenced when participants started their final fixation and ended when the putter head contacted the ball. Pre-QE was the total duration of the pre-programming and online control (Causer et al., 2017).

*Post-QE:* This was the elapsed period between when the ball had been struck and when gaze deviated away from the ball (QE-offset) by 1° of visual angle for more than 100 ms. Previous research has referred to this as QE-dwell (Causer et al., 2017).

*Total-QE:* This was calculated by measuring the elapsed period between the first frame of pre-QE and the last frame of post-QE. Literature has previously reported QE as a relative value to account for differences in trial duration (Causer et al., 2010). However, due to non-significant differences in trial duration, absolute QE values were analysed. Each participant fixated on the ball and maintained the fixation location from the pre-QE start to the post-QE end. Despite this, we did not compare gaze location or behaviour beyond the QE period as we were primarily interested in assessing the final fixation prior to shot execution.

### Statistical Analysis

The Shapiro-Wilks test of normality was used to confirm equal variance and normality of the data (p > 0.05). A x3 (skill) x4 (shot) repeated measure ANOVA were conducted for RE and trial duration. Separate x3 (skill) x4 (shot) x 9 (repetition) repeated measure ANOVAs were conducted for total-QE, pre-QE and post-QE, each shot was treated separately to measure whether the repetitions impacted how QE was applied by participants and whether a learning effect occurred. Effect sizes of main effects were calculated using Partial Eta Squared ( $\eta_{\rho }^{2})$ . To account for multiple comparisons, where appropriate, Bonferroni post-hoc analysis were used for follow up analysis, Cohen’s d effect sizes were calculated to measure the magnitude of the effect. Level of significance was accepted at p < 0.05. The relationship between the total number of successful putts and group were analysed using Chi-Square, with post-hoc analyses completed using adjusted standardised residuals.

An independent samples t-test compared the QE duration during successful putts compared to unsuccessful putts at 3m. This comparison was only conducted at 3m due to the low number of successful shots at 10m (n = 3), 12m (n = 2) and 28m (n = 0). A Cohen’s d effect size was calculated to measure the magnitude of the effect.

Pearson correlation coefficients (*r*) assessed the strength of linear relationship between total-QE duration and shot distance for sub-elite, intermediate and novice golfers. A perfect linear relationship is indicated by a *r =* 1.00, a strong correlation *r* = 0.90-0.70, a moderate relationship *r* = 0.40-0.70, a weak relationship *r* = 0.10-0.30 and no correlation *r* = 0.00 (Akoglu, 2018). Significant correlations were indicated at a p < 0.05.

Intra-rater reliability of frame-by-frame analysis was calculated through re-tracking 10% of trials (108 trials). Intra-rater reliability agreement of 99% was found, above the recommended 90% ICC threshold (Panchuk et al., 2015). This value was based upon the frame-by-frame mapping of the number of fixations (*r* = 0.97), trial duration (*r* = 0.99), relative fixation duration at the hole (*r* = 0.98) and total-QE (*r* = 0.99).

## Results

### Trial Duration

No significant main effect of shot on trail duration was observed (F = 2.206, p = 0.094, $\eta_{\rho }^{2}$ = 0.076), 3m (6.53 ± 2.14s), 10m (7.00 ± 2.25s), 12m (6.37 ± 1.99s) and 28m (6.87 ± 1.61s). A significant shot-by-group interaction was revealed (F = 2.408, p = 0.034, $\eta_{\rho }^{2}$ = 0.151), post-hoc analysis revealed no meaningful findings (see descriptives in Table 1). Trial duration of each group presented non-significant differences (F = 0.267, p = 0.768, $\eta_{\rho }^{2}$ = 0.019), as novices took 6.48 ± 1.79s, intermediates 6.56 ± 2.17s and sub-elites 7.04 ± 2.06s.

**Table 1.** Mean and Standard Deviations of Trail Duration for Each Group for all Shots Performed in the Study

	3M putt	10M putt	12M chip	28M chip
Novice (s)	6.48 ± 2.09	6.22 ± 1.77	6.04 ± 1.47	7.17 ± 1.56
Intermediate (s)	6.51 ± 2.33	7.43 ± 2.70	5.79 ± 1.27	6.50 ± 1.77
Sub-elite (s)	6.59 ± 2.00	7.35 ± 1.96	7.28 ± 2.61	6.92 ± 1.40

### Shot Success

At 3m, novice, intermediate and sub-elite groups completed 9, 23 and 25 successful putts, respectively. At 10 m, novice, intermediate and sub-elite groups completed 0, 1 and 2 successful putts, respectively. At 12 m, all groups achieved 2 successful chips. No successful chips were recorded in any group at the 28 m chip distance.

With the low number of successful shots completed at 10 m, 12 m and 28 m distances, success was only assessed when putting from 3m. There was a significant association between expertise and number of successful putts χ^2^ (2) = 8.00, p = .02, with post-hoc testing identifying that both sub-elite and intermediate groups were more likely to complete successful putts.

#### Successful vs. Unsuccessful Putts

At 3m there were no significant differences in the duration of total QE for successful putts (2.66 ± 1.10s) compared to unsuccessful putts (2.51 ± 1.13s) (t (268) = 0.907, p = 0.365, d = 0.135).

### Radial Error 

**Figure 2.**
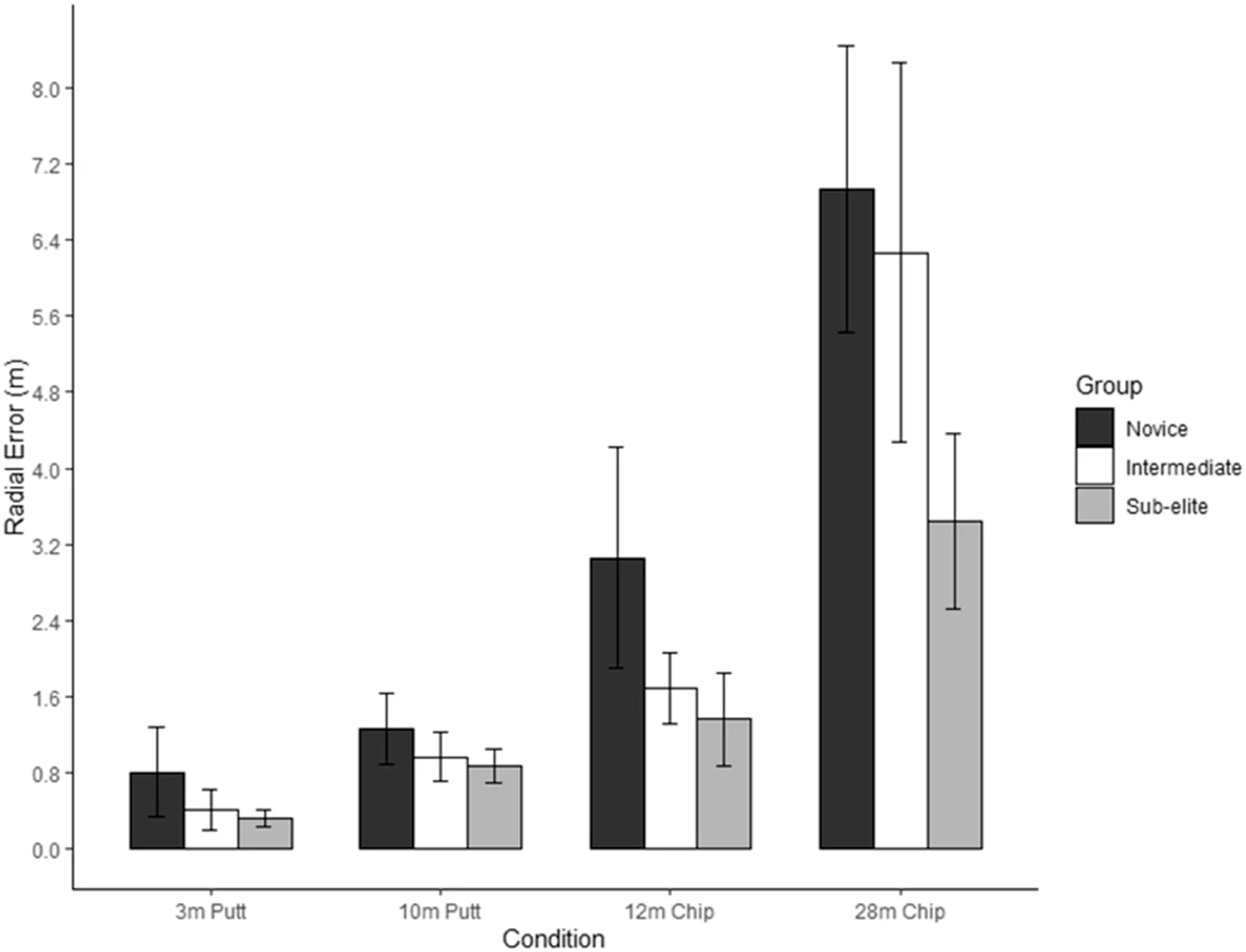
RE (m) for Each Level of Expertise at all Distances Assessed

A significant main effect of shot on performance was observed (3m; 0.51 ± 0.36 m, 10m; 1.03 ± 0.32 m, 12m; 2.04 ± 1.03, 28m; 5.55 ± 2.11 m) (F = 213.109, p < 0.001, $\eta_{\rho }^{2}$ = 0.888) and group (novices; 3.02 ± 2.60 m, intermediate; 2.33 ± 2.51 m, sub-elite; 1.50 ± 1.29 m) (F = 24.554, p < 0.001, $\eta_{\rho }^{2}$ = 0.645). A significant shot-by-group effect was observed (F = 9.453, p < 0.001, $\eta_{\rho }^{2}$ = 0.412), see Figure 2 for shot-by group comparisons. Post-hoc analysis indicated that sub-elite and intermediate groups had lower RE compared to novices at 3m (p = 0.001, d = 1.516. p = 0.008, d = 1.117), 10m (p = 0.004, d = 1.431. P = 0.024, d = 0.995), and 12m (p < 0.001, d = 2.007. p < 0.001, d = 1.685). The sub-elite group has significantly lower RE compared to novice (p < 0.001, d = 2.947) and intermediate (p < 0.001, d = 1.919) groups at 28m. RE within the novice and intermediate groups significantly increased as the distance became greater, whereas sub-elite RE increased from 3m to 10m (p < 0.001, d = 4.048) and 12m to 28m (p < 0.001, d = 4.050), with no difference between the 10m and 12m distance (p = 0.074, d = 1.418). See Figure 2 for performance at each level of expertise at all distances assessed.

### Quiet Eye 

#### Total-QE

**Figure 3.**
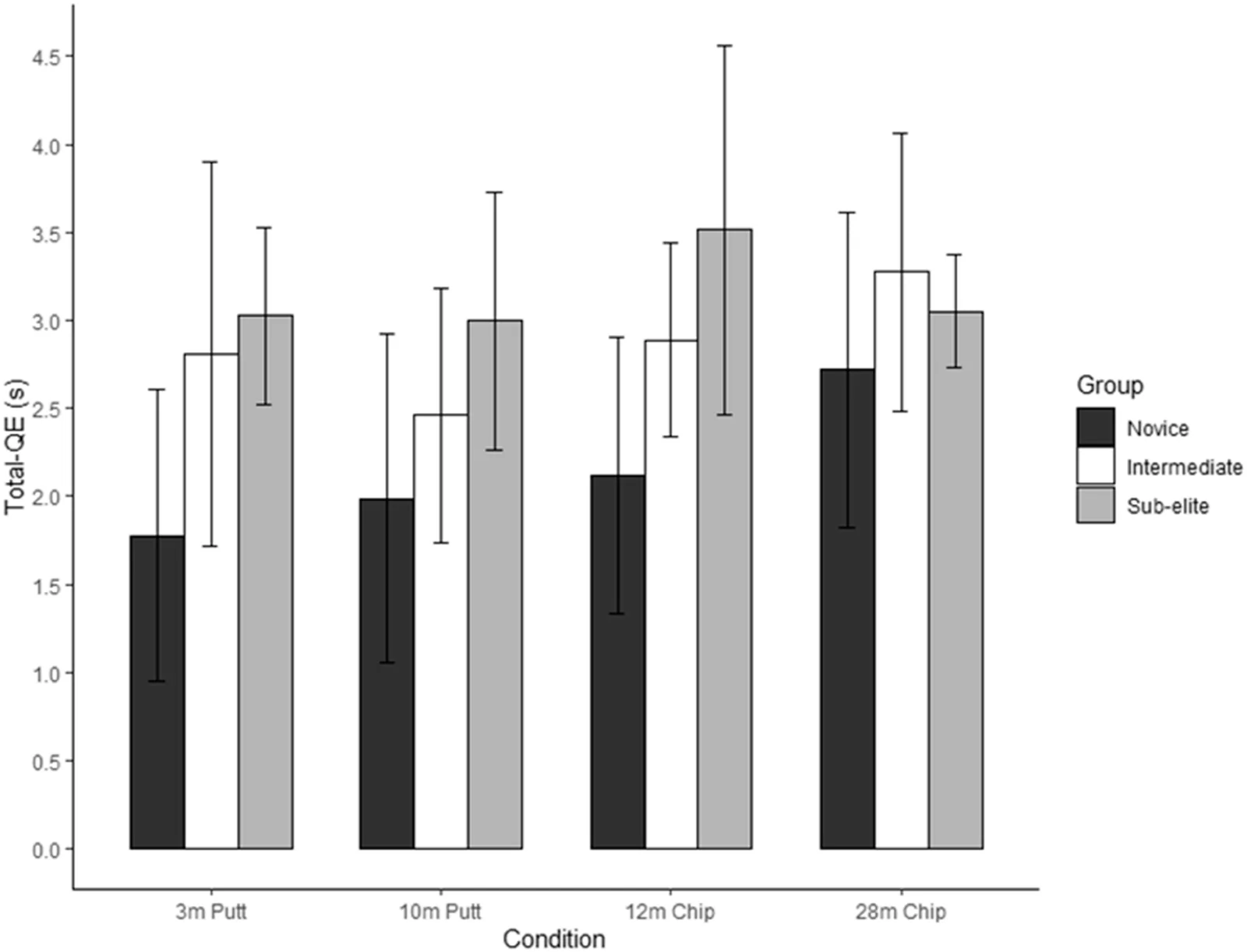
Total-QE (s) for Each Level of Expertise at all Distances Assessed

A significant main effect of shot on total-QE was observed (3m; 2.54 ± 0.97,10m; 2.48 ± 0.86s, 12m; 2.84 ± 0.97s, 28m; 3.01 ± 0.72s, F = 4.773, p = 0.004, $\eta_{\rho }^{2}$ = 0.150), post-hoc analysis indicated that participants implemented significantly longer total-QE when performing the 28m shot compared to putting from 3m (p = 0.011, d = 0.559) and 10m (p = 0.002, d = 0.672). There was a non-significant shot-by-group interaction effect (F = 1.802, p = 0.109, $\eta_{\rho }^{2}$ = 0.118), see Figure 3 for shot-by-group comparisons. There was a main effect for group (F = 7.874, p = 0.002, $\eta_{\rho }^{2}$ = 0.368), post-hoc analysis revealed that sub-elites (3.15 ± 0.70s, p < 0.001, d = 1.308) and intermediates (2.86 ± 0.82s, p = 0.011, d = 0.863) implemented a significantly longer total-QE compared to novices (2.15 ± 0.82s). There was a non-significant repetition main effect for total-QE (F = 0.555, p = 0.814, $\eta_{\rho }^{2}$ = 0.020).

#### Pre-QE

**Figure 4.**
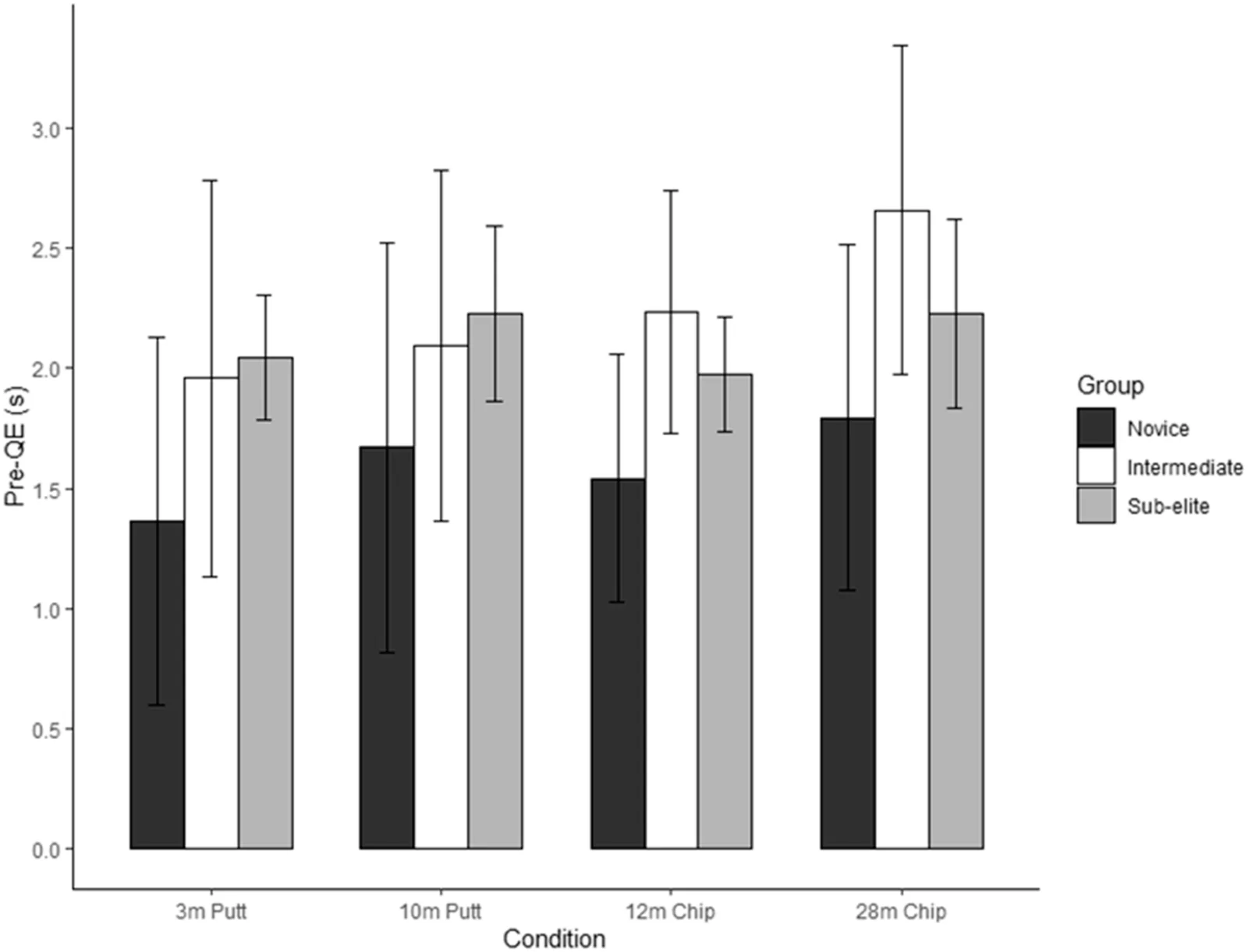
Pre-QE (s) for Each Level of Expertise at all Distances Assessed

A significant main effect of shot on pre-QE was observed (F = 6.613, p < 0.001, $\eta_{\rho }^{2}$ = 0.197, 3m; 1.79 ± 0.07,10m; 2.00 ± 0.69, 12m; 1.92 ± 0.51, 28m; 2.23 ± 0.68), post-hoc analysis revealed that golfers implemented longer pre-QE periods at 28m compared to 3m (p < 0.001, d = 0.630), 10m (p = 0.037, d = 0.135) and 12m (p = 0.001, d = 0.517), pre-QE was significantly longer during the 10m putt compared to the 3m putt (p = 0.005, d = 0.298). No shot-by-group interaction effect was present (F = 1.342, p = 0.248, $\eta_{\rho }^{2}=$ 0.090). See Figure 4 for shot-by group comparisons. Analysis indicated a main effect for group (novices; 1.59 ± 0.71s, intermediates; 2.24 ± 0.71s, sub-elite; 2.12 ± 0.33s) (F = 4.613, p = 0.019 $\eta_{\rho }^{2}$ = 0.255), pre-QE duration for sub-elites (p = 0.027, d = 0.957) and intermediates (p = 0.008, d = 0.915) were significantly longer than novices. There was a non-significant repetition main effect for pre-QE (F = 0.154, p = 0.145, $\eta_{\rho }^{2}$ = 0.054).

**Figure 5.**
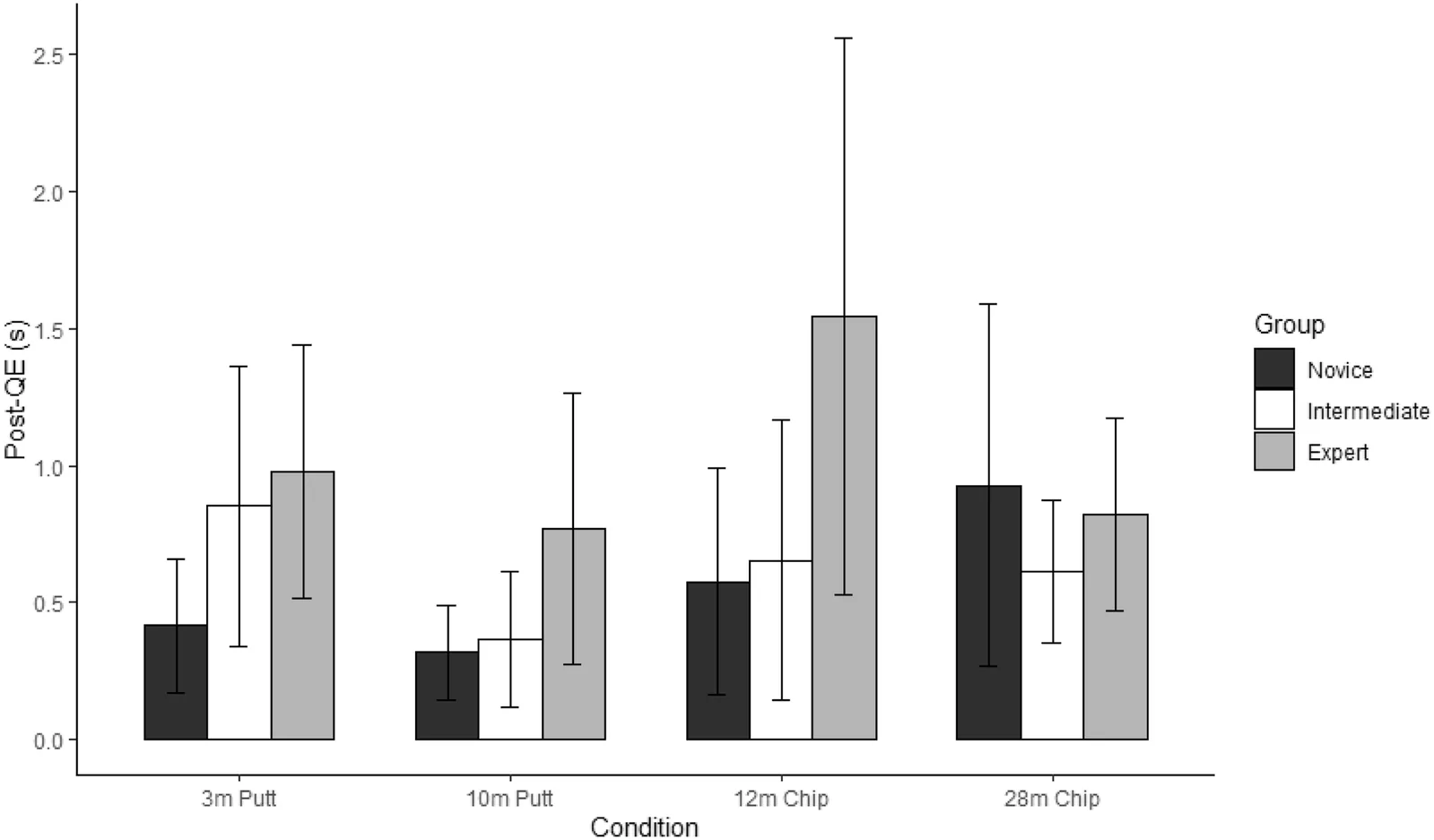
Post-QE (s) for Each Level of Expertise at all Distances Assessed

***Post-QE.*** A significant main effect of shot was observed on post-QE (F = 5.303, p = 0.002, $\eta_{\rho }^{2}$ = 0.164, 3m; 0.75 ± 0.47,10m; 0.48 ± 0.98, 12m; 0.93 ± 0.38, 28m; 0.79 ± 0.45). There was a significant shot-by-group interaction effect (F = 3.531, p = 0.004, $\eta_{\rho }^{2}$ = 0.207), sub-elites (p = 0.006, d = 1.608) and intermediates (p = 0.028, d = 1.153) at 3m implemented a significantly longer post-QE than novices. At 10 m and 12m sub-elites post-QE was significantly longer compared to intermediates (10m; p = 0.012, d = 1.083. 12m; p = 0.008, d = 1.172) and novices (10m; p = 0.006, d = 1.284. 12m; p = 0.004, d = 1.322), at 28m there were no significant differences. See Figure 5 for shot-by group comparisons. A main effect for group was present (novices; 0.56 ± 0.42s, intermediates; 0.62 ± 0.42s, sub-elites 1.03 ± 0.67) (F = 6.403, p = 0.005, $\eta_{\rho }^{2}$ = 0.322). There was a non-significant repetition main effect for post-QE (F = 0.377, p = 0.932, $\eta_{\rho }^{2}$ = 0.014).

### Pearson Correlation Coefficient

Total-QE of novice golfers indicated a significant linear relationship with shot complexity (*r* = 0.393, p=<0.012). Total-QE of intermediates displayed a moderate non-significant linear relationship with complexity (*r* = 0.261, p = 0.103). Total-QE and task complexity of sub-elite golfers did not indicate a linear relationship between variables (*r* = −0.005, p = 0.974).

## Discussion

This study examined the impact of expertise and task complexity on the accuracy of golf shots and pre, post and total-QE. Sub-elite and intermediate golfers performed better than novices in simpler tasks. However, sub-elite golfers performed better than intermediates (and novice) in the most complex condition. Irrespective of complexity, sub-elites and intermediates implemented longer pre-QE and total-QE periods than novices. Intermediate and novice post-QE continually increased, resulting in no group differences being observed within the most complex condition. Similar trends were observed when assessing the relationship of each groups task complexity and total-QE duration, novice and intermediate golfers continually extended their QE whereas sub-elite golfers implemented similar durations irrespective of complexity. This suggests a maximum QE period that benefits performance and supports the notion that extensively long QEs may be counterproductive (Janelle et al., 2000; Klostermann et al., 2018). Moreover, novices seemed to focus on visual-spatial processing and accrue additional visual feedback, indicated through a gradual extension of the QE period as complexity increased, indicating a linear relationship. Irrespective of task complexity, sub-elite QE displayed greater consistency throughout each condition, indicating that this period may be used for a similar purpose (to parameterise optimal movement execution) (Klostermann et al., 2018). Therefore, the mechanisms related to the QE period may alter as an individual’s skill increases.

In accordance with previous research, longer QE periods observed in sub-elite and intermediate golfers coincided with better performance (Vickers, 1992, 1996; Williams et al., 2002), which was indicated by participants hitting the ball closer to the hole (reduced RE) and smaller standard deviations, which suggests greater consistency. This extended period likely allowed the prioritisation of task-salient cues, possibly resulting in the cortical resources being reallocated away from analytical processing and irrelevant cues to allow effective motor programming and execution (Mann et al., 2016). This desynchronisation (reduction) of cognitive processes causes a dampening effect on brain activity (Mann et al., 2016) and supports the neural efficiency hypothesis, which suggests a more efficient control of cortisol, as skilled performers evidenced a greater inhibition of occipital alpha power response (this has also shown to decrease prior to poorer performance) and lower the amplitude of activity observed through fMRI-BLOD activity compared to less skilled individuals (Del Percio et al., 2009). Moreover, training has induced reductions in motor cortex activity, displaying suppression of cognitive processes (Del Percio et al., 2009). These effects contribute to a desynchronisation (reduction) of cognitive processes, facilitating more accurate golf shots (lower RE). Research has demonstrated that a reduced QE variability is a strong predictor of performance in aiming tasks (Mizusaki et al., 2025), consistently implementing similar QE durations likely occurs from training and experience, which contributes to higher-skilled performers attaining a superior state of mental readiness more frequently in comparison to less skilled counterparts. This contributes to a consistent, more efficient strategy due to reduced brain activity (and brain regions activated), which may be attributed to sub-elite and intermediate golfers finding the tasks less effortful than novices. Interestingly, the effect of complexity did not alter QE durations during more difficult conditions, and a ceiling effect was observed.

Our findings indicate that novice golfers gradually extended their QE as complexity increased, evidenced by a 53% increase between the simplest and most complex conditions, whereas a 1% difference was observed within sub-elites. This indicates that QE within novice golfers is sensitive to task demands and that the equivalent cognitive load placed may not be experienced within sub-elite golfers. Less skilled athletes have been shown to implement more fixations (Williams et al., 2002), which could result in the accruement of additional task-irrelevant visual information, which may lead to extension of the QE period and contributes to a linear relationship. Through acquiring task-irrelevant information novices are more likely to exhibit uncertainty and hesitation, which would likely lead to greater cortical activation, which has been associated with poorer preparation (Carey et al., 2024). These results confirm our hypothesis that novice QE increases with task complexity. This relationship is not observed in sub-elite golfers, which supports the proposal that there is a maximal QE length that offers performance benefits and infers that excessively long QE periods are counterproductive (Janelle et al., 2000; Klostermann et al., 2018). Alternatively intermediate QE increased by 16% between the simplest and most complex conditions, Although, an increase was observed, this did not represent a significant correlation, it may be that intermediates utilise the QE similarly to sub-elite golfers but are unable to replicate performance in more challenging conditions due to a less efficient QE and possibly poorer technique. Collectively, this suggests a ceiling effect.

A ceiling effect may indicate that that purpose of the QE for skilled golfers is independent of task difficulty. Despite an internal focus of attention (force and technical aspects of the shot) being negatively associated with performance (Parr et al., 2023) recent multi-measure studies have highlighted the mechanisms underlying the QE period are likely related to movement planning (Carey et al., 2024; Gallicchio & Ring, 2020). This provides support for the inhibition hypothesis which suggests longer QE durations occur when skilled individuals eliminate suboptimal movement variants to ensure the optimal movement variant is parameterised (which occurs during preparation and execution) (Klostermann et al., 2014). This explanation is only valid to the sub-elite group who reported greater experience and were likely to have been exposed to multiple movement variants from previous playing environments. The non-linear relationship supports this hypothesis as the QE period must be independent of cognitive load and visual processing if consistent periods are being implemented. Additionally it has been suggested that golfers may process visual information during the green reading phase and rely on working memory to ensure that they can effectively prepare for the movement during the QE (Gallicchio & Ring, 2020) which would allow golfers to predominantly focus on technical execution, such as postural stability and quality of ball-strike. As information is processed prior to putt initiation it is likely to lead to a better preparation through a greater suppression of cortical activity which has been associated with successful golf performance (Carey et al., 2024). Contrary to this, Mann et al. (2011) reported that cortical activity of higher skilled golfers highlighted a primary focus on visual-spatial processing at a putting distance of 3.6 m; however, this single task study did not assess whether this would alter as task demands require greater technical skill. Our findings likely support that the mechanisms underlying the QE period are primarily focusing on movement-related processes as the QE of sub-elite golfers did not increase, despite a gradual increase in task demands, and therefore we propose that the purpose of the QE period is consistent and is not impacted by the difficulty of the task in skilled performers.

Irrespective of group, it cannot be ignored that QE durations increased in the most complex task compared to putting, which supports that QE represents a pre-programming period (Mann et al., 2011; Williams et al., 2002). This effect was likely driven by the increase in QE observed in novices and intermediates, which supports the possibility that the QE period represents a different purpose for contrasting levels of expertise and indicates a transition from processing information and the accruement of feedback (novice/intermediates) to the selection of optimal movement variants alongside the processing of visual information (sub-elites). This transition likely occurs due to greater experience and training adaptations within the underlying mechanisms of QE. To attain optimal performance, athletes must be able to parameterise optimal movement variants (Klostermann et al., 2018), process visual information prior to task initiation (Mann et al., 2011) and utilise visual and/or proprioceptive feedback (Causer et al., 2017). It must be acknowledged that research has indicated the QE duration is sensitive to task complexity (Williams et al., 2002), type of motor skill (Klostermann et al., 2014, 2018; Vickers, 1996) and contextual factors (Runswick et al., 2021); as an optimal QE length is likely task dependent (Harris et al., 2023) the same ceiling effect (i.e., the absolute value) may not transfer across all tasks and sports.

Regardless of shot distance novices did not perform at a similar level as the sub-elite group, even when QE was not significantly different between groups. These findings support the assumption that performance’s relationship with QE is not linear in sub-elite golfers and that a ceiling effect may be a distinguishing factor between levels of expertise. This inverted-U relationship may be an accurate representation (Klostermann et al., 2018) for novices, however sub-elite and skilled golfers likely implement a consistent (maximum) QE duration that is maintained irrespective of task demands. It has been proposed that optimal performance corresponds to a QE period of 3063 ms when completing a throwing task (Klostermann et al., 2018); this is similar to the QE durations that were observed within sub-elite golfers (mean 3147 ms) in the present study. Shorter QE periods may have contributed to poorer performance through a sub-optimal preparatory period. Similarly to previous research, the longest QE periods in the present study were in the most difficult condition, which represents enhanced task demands in relation to movement parameters (force, velocity and direction and the requirement to process additional visual cues (Causer et al., 2017; Williams et al., 2002). For instance, novice golfers (Causer et al., 2017) reported an increased pre-programming duration as putting distance increased when comparing 1.83 m putts to 3.35 m. This linear effect was similarly identified in the work of Williams et al. (2002), where QE durations increased irrespective of expertise, even when comparing skilled and less skilled billiard players. These findings indicate that QE period is likely to be used to process and programme visual cues prior to movement initiation, however we propose that this is likely attributed to less skilled individuals and that higher skilled golfers primarily process movement-related information. Therefore the QE period likely represents several hypotheses presented by (Gonzalez et al., 2017) and that the purpose/mechanisms underlying the QE may alter as an individual’s ability increases.

Interestingly, there were non-significant interaction effects observed for total-QE and pre-QE. This indicates that the trends observed were consistent across condition and expertise. However, there was a significant post-QE interaction effect was present, suggesting greater sensitivity to task demands and expertise. These findings are likely due to the conflicting purpose of each phase. Pre-QE is associated with processing of visual cues and optimal movement parameterisation (Carey et al., 2024; Gallicchio & Ring, 2020). Therefore, the processes that occur during pre-QE are likely more impactful on performance, as the effect of contrasting instructional guidance revealed that QE-offset (after movement initiation) was the most critical to optimise performance due to incorporating online control processes (Klostermann et al., 2014). As we conducted an observational study and aimed to compare QE pre and post execution, the online control processes were accounted for during pre-QE in the present study; therefore, the processes that occur prior to task execution are likely the most critical for superior performance. This may explain why pre-QE and total-QE results were similar, and that pre-QE drives this effect. The importance of pre-QE is further supported as longer total-QE and pre-QE coincided with expertise-based performance differences (shot accuracy). Despite pre-QE having a greater contribution to task execution, post-QE may aid future performance as it is associated with multiple feedback streams (proprioceptive and visual feedback) (Causer et al., 2017), which may guide future actions in golf, however, the need to have an extended post-QE may be negligible for the current shot being performed. Previous work has suggested that a shorter post-QE may indicate attention being diverted away from the ball prematurely and was associated with unsuccessful putts (Vine et al., 2011). It is plausible that if the acquired feedback during shot execution is likely to lead to poor performance, then golfers may deviate fixation location earlier. Therefore, longer sub-elite post-QE may indicate quality of shot execution, whilst allowing golfers to acquire feedback that guides planning for future shots.

The present study can be used to verify previous research examining expertise and task complexities’ impact on QE. Typically, studies assessing golf putting have been conducted in a laboratory setting (Causer et al., 2017; Klostermann et al., 2018; Lier, 2011; Mann et al., 2011; Vine et al., 2011; Wilson & Pearcy, 2009). A notable difference observed within this study was the elongated QE periods (sub-elite QE approx. 3s). Previous research comparing similar putting distances presented QE durations between 0.86-2.2s (Carey et al., 2024; Causer et al., 2017; He et al., 2024; Klostermann et al., 2014; Walters-Symons et al., 2017). However, through assessing golfers within their natural competitive environment, and accounting for the effect of gradient, wind, imperfect putting surfaces and other environmental distractions, the study holds robust findings due to the study design contributing to a greater ecological validity (Kihlstrom, 2021). Therefore, irrespective of the mechanisms underpinning the QE period being inward (pre-programming) or outward (attentional control/motor planning) functions described by (Harris et al., 2023), golfers within their typical environment require an additional period to attain mental readiness. Despite this, the present study confirms that the expertise trends typically observed within laboratory-based studies are transferable to the environment that golfers compete within (Causer et al., 2017; Klostermann et al., 2014; Mann et al., 2011; Vine et al., 2011).

Inconsistent findings have been presented when assessing the association between QE durations and golf performance, with studies reporting longer QE durations during successful putts (Lier, 2011; Mann et al., 2011; Wilson & Pearcy, 2009) and others reporting similar QE durations irrespective of shot success (Carey et al., 2024; Ziv & Lidor, 2019). Our findings did not detect QE duration differences as a function of shot success at 3m. Carey et al. (2024) identified distinctly different neural processes underlying successful and unsuccessful putting despite a similar QE length; therefore, this may indicate that, in golf, the purpose of the attentional networks is to strike the ball optimally (through optimising movement patterns), if this is the case, then measuring success may not adequately measure skill execution. Performance is multifaceted; therefore, using binary outcomes to measure performance may neglect key differences in QE profiles. In the current study the primary measure of performance was shot accuracy, indicated by RE. The purpose of using this measure was to ensure complexity (and shot type) could be manipulated beyond previous research (Causer et al., 2017; Klostermann et al., 2014; Lier et al., 2010; Walters-Symons et al., 2017; Wilson & Pearcy, 2009). Although the number of successful shots was limited at 10m, 12m and 28m, a golfer’s ability to be more accurate is integral for competition outcome. Moreover, within in-situ study designs, environmental factors outside a golfer’s control may impact the outcome despite the individual executing the shot as intended. Therefore, future laboratory and in-situ study designs may incorporate additional analyses and assess QE’s relationship with factors such as shot accuracy (radial error), kinematic assessments (club head velocity and angle) and neural networks. Additionally, the variety of definitions of expertise and measures of performance may have impacted the consistency of findings in the literature. Future studies should consistently and objectively define levels of expertise, for example, by using the scale developed by Swann et al. (2015).

Our findings may have implications when considering QE training interventions. As sub-elite golfers implemented consistent QE durations, the primary aim of an intervention must be to ensure consistency in the duration and that this is robust in competitive environments. Moreover, practitioners may look to focus on the duration of the QE period prior to the ball being struck, as our findings suggest that this is the phase of QE that contributes most to performance, evidenced by increased putting accuracy (lower RE). Alternatively, where necessary, if golfers were implementing excessively long QE periods, there may be a need to reduce these to a more optimal range to avoid distractions. The effect of QE interventions within elite golfers has indicated an extension of QE during retention and pressure tests, the opposite effect to the control group (Vine et al., 2011), possibly leading to a sub-optimal QE duration, which will likely impact suppression of cognitive processes (Mann et al., 2016). These have shown promise in managing anxiousness, with elite golfers reporting less anxiety following QE interventions (He et al., 2024). Therefore, manipulation of pressure or anxiety must be incorporated to measure the efficacy of such interventions in elite golfers. For less skilled golfers, the aim must be to extend the QE period to allow the quieting of cognitive processes. Despite QE interventions being provided to novice golfers, Vine and Wilson (2010) reported that QE periods three times longer than pre-intervention did not improve performance compared to the technically trained group or the learning rate. This highlights that combining technical and QE intervention within a novice population may be beneficial, or delivery of cognitive interventions to golfers following technical training could lead to greater results. Therefore, QE interventions may differ as a function of expertise, highlighting the importance of a holistic approach and individual-specific training regimes. Moreover, the importance of this is also evident from the variability of performance and QE durations within each group. To control for intragroup variability, researchers have measured both inter- and intra-group differences. It has been shown that better golfers implement longer QE periods on successful shots, although this did not occur for every higher-skilled golfer (Mann et al., 2011). Those delivering QE interventions must gain a thorough understanding of QE and the performance profile of an individual athlete prior to intervention to ensure effective training plans are developed and delivered.

Despite examining golfers in a naturalistic environment, the tasks selected in the study did not appear to alter the cognitive load within sub-elite golfers. Contrary to this effect, the cognitive load in novices appeared to increase with complexity; the expertise-QE correlations indicated these effects. Researchers may want to explore if this is a consistent feature within those at higher levels of expertise and whether this behaviour transfers to a competitive environment. Cognitive load is the allocation of mental resources invested into the resolution of a task, which can be impacted by complexity and emotional regulation (Fuster et al., 2021). Cognitive load can be maintained if an athlete does not allocate more effort to a task, which may explain the consistent QE periods of sub-elite golfers. This effect could be interpreted to be the expertise reversal effect presented in the cognitive load theory (Runswick et al., 2018). Over time, sub-elite golfers develop an ability to automatically integrate extra task-relevant information using an existing schemata (automation of information processing), whereas these automatic processes in individuals with limited exposure to the sport are likely underdeveloped. Previous studies have assessed cognitive load by assessing pupillary response during a sporting action, which was beyond the scope of this study. We did not measure the neural activity, which has been previously conducted within a laboratory setting (Carey et al., 2024; Mann et al., 2011), manipulate anxiety, or the context under which shots were performed, which has been shown to impact QE alongside performance (Giancamilli et al., 2022; He et al., 2024; Runswick et al., 2021). Therefore, we cannot explicitly identify the mechanism underpinning our findings and the QE period. Future research may explore the QE, neural activity and pupillary response between multiple conditions where complexity and/or cognitive load is manipulated; these multi-measure approaches could aid the identification of the mechanisms underpinning the QE (Carey et al., 2024). Additionally, sub-elite and intermediate golfers were instructed to use identical clubs to novices when chipping; this may not have corresponded to the club they would have normally selected, which may have impacted performance. However, this study explored QE in a naturalistic environment, verifying and examining multiple levels of expertise across contrasting difficulty levels.

To summarise, sub-elite golfers implemented similar QE durations throughout each task, supporting the inhibition hypothesis (Klostermann et al., 2018). As novices progressed into the most complex condition, their QE increased by 53% which may indicate that novices use the QE period predominantly for pre-programming (Causer et al., 2017; Williams et al., 2002), whereas sub-elites likely use this to select and prepare the optimal movement variant to ensure optimised performance, evidenced through the application of a consistent QE duration. Sub-elite golfers likely process visual information in the green reading phase (prior to task execution), which enables parameterisation of optimal movement patterns to occur during the QE period. Although intermediate QE increased 16%, between simplest to hardest condition, this did not present a significant correlation, it may be that intermediates utilise similar mechanism to sub-elite golfers during the preparatory phase, but are less efficient and technically poorer contributing to inferior performance. The study suggests that beyond a certain maximum duration, the continuation of QE ceases to have a positive effect on performance, indicated by our analysis of expertise-based differences. A maximal duration contributes to superior performance when considering both success and accuracy (RE) in golf. This maximal duration will likely differ between individuals; however, our findings were similar to the 3000 ms threshold suggested by Klostermann et al. (2018). The optimal duration may be sensitive to task demands (complexity, contextual, and cognitive demands), particularly for less-skilled golfers. However, the mechanisms that underpin the QE period are evidently a distinguishing factor in the performance of aiming targets in golf (putting and chipping).

## Data Availability

Data associated with this project can be found at: https://osf.io/jxwka/?view_only=5564b02a922943dd82c2979e81374195.
